# Redox Linked Flavin Sites in Extracellular Decaheme Proteins Involved in Microbe-Mineral Electron Transfer.

**DOI:** 10.1038/srep11677

**Published:** 2015-07-01

**Authors:** Marcus J. Edwards, Gaye F. White, Michael Norman, Alice Tome-Fernandez, Emma Ainsworth, Liang Shi, Jim K. Fredrickson, John M. Zachara, Julea N. Butt, David J. Richardson, Thomas A. Clarke

**Affiliations:** 1 Centre for Molecular and Structural Biochemistry, School of Biological Sciences and School of Chemistry, University of East Anglia, Norwich NR4 7TJ, United Kingdom; 2 Pacific Northwest National Laboratory, Richland, WA 99352, USA

## Abstract

Extracellular microbe-mineral electron transfer is a major driving force for the oxidation of organic carbon in many subsurface environments. Extracellular multi-heme cytochromes of the *Shewenella* genus play a major role in this process but the mechanism of electron exchange at the interface between cytochrome and acceptor is widely debated. The 1.8 Å x-ray crystal structure of the decaheme MtrC revealed a highly conserved CX_8_C disulfide that, when substituted for AX_8_A, severely compromised the ability of *S. oneidensis* to grow under aerobic conditions. Reductive cleavage of the disulfide in the presence of flavin mononucleotide (FMN) resulted in the reversible formation of a stable flavocytochrome. Similar results were also observed with other decaheme cytochromes, OmcA, MtrF and UndA. The data suggest that these decaheme cytochromes can transition between highly reactive flavocytochromes or less reactive cytochromes, and that this transition is controlled by a redox active disulfide that responds to the presence of oxygen.

Many species of Gram-negative bacteria are capable of coupling anaerobic growth to the extracellular respiration of insoluble minerals containing Fe(III) and Mn(IV) or artificial electrodes. This process requires that electrons released during intracellular oxidative catabolic reactions be transported across the cellular outer membrane and then from the cell surface to the terminal electron acceptor^1^. One of the best-studied genera of mineral-respiring bacteria are the *Shewanellacea;* these are Gram-negative facultative anaerobes found globally in aquatic sediments that can be cultured either in a planktonic form or as a biofilm on the surface of insoluble minerals or electrodes^2^. Intensive study of the interactions between *Shewanella oneidensis* MR-1 and both soluble and insoluble metal oxides has lead to four proposed mechanisms that could allow *S. oneidensis* to transfer electrons from cell surface to extracellular acceptor. Two involve direct electron transfer either by direct contact between microbe and mineral or through conductive extended nanowires^3^,^4^,^5^,^6^. The other two involve mediated electron transfer through soluble electron shuttles such as chelated metal ions or secreted flavin molecules^7^,^8^.

The genome of *S. oneidensis* contains a metal-respiring operon (*mtr*) that is essential for reduction of both soluble and insoluble metal ions^2^. The *mtr* operon includes the genes to express three proteins: a decaheme cytochrome MtrA that inserts into the periplasmic side of a 28-strand transmembrane β-barrel MtrB^9^, and an outer membrane decaheme cytochrome MtrC that inserts into the extracellular side of MtrB. Multiple lines of evidence indicate that electrons can be exchanged directly between the hemes of both MtrC and MtrA within MtrB^10^. This MtrCAB ‘porin cytochrome’ complex has been shown to transfer electrons directly to insoluble metal oxides when inserted into liposomes and provided with an electrochemical force in the form of reduced methyl viologen, suggesting it is possible for mineral oxides to be directly reduced by the MtrCAB complex^4^. In addition to the *mtrCAB* genes, the metal-respiring locus also contains the gene for a second outer membrane cytochrome *omcA* and an *mtrDEF* gene cluster paralogous to the *mtrCAB* gene cluster, however the expression of these genes are under separate promotors^2^. The outer membrane cytochromes MtrC, OmcA and MtrF are exported to the extracellular cell surface by the type II secretion system^11^ and *S. oneidensis* Δ*omcA-*Δ*mtrC* double mutants are severely compromised for respiratory mineral Fe(III) reduction and electron transfer to anodes in microbial fuel cells.

The sequenced genomes of different *Shewanella* species contain a range of these outer membrane multiheme cytochromes (OMMC), which can be phylogenetically organised into the four clades MtrC, OmcA, UndA and MtrF^12^. The OMMC of the MtrC and OmcA clades are the most widely studied^13^,^14^,^15^,^16^; the UndA clade members are OmcA homologues found in a range of *Shewanella* species, while the MtrF clade members are paralogues of MtrC.

The molecular structures of three OMMCs have been determined: the deca-heme MtrF and OmcA of *S. oneidensis* and the undeca-heme *S. HRCR-6* UndA^17^,^18^,^19^. All structures are formed of 4 domains, two multiheme domains that are flanked by two β-barrels with β-strands arranged in Greek key motifs. Both MtrF and OmcA contain a conserved deca-heme ‘staggered cross’ cofactor arrangement, with UndA containing an eleventh heme that is inserted between hemes 6 and hemes 7 in the amino acid sequence. The staggered-cross heme arrangement means there are four potential sites for electrons to enter and exit the structure, with two opposing ends of the cross pointing into the β-barrels and two exposed at the edges of the multiheme domains. The structures of OmcA, UndA and MtrF also revealed a conserved CX_8–15_C disulfide within the β-barrel of domain III. UndA and OmcA also contain a second CX_2-3_C disulfide within the N-terminal β-barrel domain I, this CX_3_C motif is present in the amino acid sequence of MtrF, but the putative disulfide bond was not resolved due to poor electron density in that domain^17^,^18^,^19^.

There is clear evidence that *S. oneidensis* secretes both riboflavin and flavin mononucleotide (FMN) and that these secreted flavins have a substantial impact on the ability of *S. oneidensis* to reduce Fe(III)oxides^7^,^20^. One hypothesis is that both Riboflavin and FMN function as soluble redox mediators that facilitate electron exchange between *S. oneidensis* and solid metal oxides. This is supported by studies that identified a *bfe* gene in *S. oneidensis* that was essential for secretion of flavin into the extracellular medium, deletion of this gene caused a 75% decrease in the ability of *S. oneidensis* to reduce Fe(III)oxides or transfer electrons to graphite electrodes. As both *bfe* and *mtr* are important for extracellular electron transfer it is likely that flavins must interact with either directly or indirectly with extracellular outer membrane multiheme cytochromes (OMMC) on the cell surface^21^. Paquete and co-workers used NMR to measure dissociation constants of 29 μM and 259 μM between oxidised FMN and oxidised MtrC or OmcA respectively. Given that the *S. oneidensis* extracellular FMN concentration does not exceed 1 μM during growth, these dissociation constants suggest a transient interaction between FMN and the OMMC^22^. However, electrochemical and voltammetric studies on *S. oneidensis* biofilms generated on the surface of electrodes indicated that under anaerobic conditions MtrC associated with FMN to produce a semi-reduced flavin at the biofilm-flavin interface, suggesting the formation of a MtrC-FMN complex^23^. These different results could be harmonised if the interaction between MtrC and FMN was different under aerobic and anaerobic respiratory conditions. Here we demonstrate a reversible transition of MtrC between cytochrome and flavocytochrome states that is controlled by the redox state of a conserved disulphide. *S. oneidensis* strains that are unable to form the disulfide are severely compromised in their ability to grow aerobically, but not anaerobically, suggesting that the MtrC-FMN flavocytochrome may reduce oxygen and produce reactive oxygen species, so its formation must be closely regulated during life at oxic-anoxic interfaces.

## Results

### The S. oneidensis MtrC CX_8_C motif is required for aerobic growth

**Figure 1:**
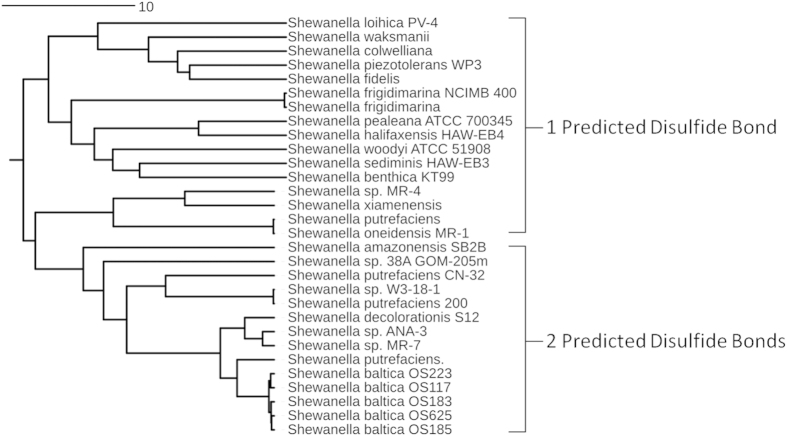
Phylogenetic alignment of the currently available amino acid sequences of MtrC from different *Shewanella* species. Alignment of sequences based on homology reveals that *Shewanella* can be arranged into two groups on the basis of the number of predicted disulfide bonds.

Comparative amino acid sequence analysis of the members of the currently available OMMC clades reveals that all OMMC contain a highly conserved CX_8–15_C within the sequence corresponding to domain III^17^. MtrF, UndA and OmcA also contain a second CX_2-3_C motif within the N-terminal domain I. However, the MtrC OMMC clade is spilt into two sub-clades groups; the first MtrC group, MtrC1, contains two CX_5–8_C motifs while the second, MtrC2, contains just a single CX_8_C motif in the sequence corresponding to domain III and is the group that contains *S. oneidensis* MR-1 (Fig. 1). Currently, the function of the non-conserved CX_2-3_C motif is not known, but the presence of just one CX_8_C motif in *S. oneidensis* MR-1 MtrC would make it the enzyme of choice for functional studies on the role of this conserved feature.

A recombinant MtrC expression system in *S. oneidensis* LS661 was generated as described in methods. This *S. oneidensis* strain lacked the genomic copy of *mtrC,* but contained an inducible *mtrC* gene on a pBAD expression vector (pLS172) instead. In order to replace the MtrC cysteine residues Cys_444_ and Cys_453_ with alanines, site directed mutagenesis was used to modify pLS172 into pLS172-C444A, C453A, which was transformed into LS661 to give *S. oneidensis* LS661, pLS172-C444A, C453A. Expression of the recombinant *mtrC* gene was induced by arabinose.

**Figure 2:**
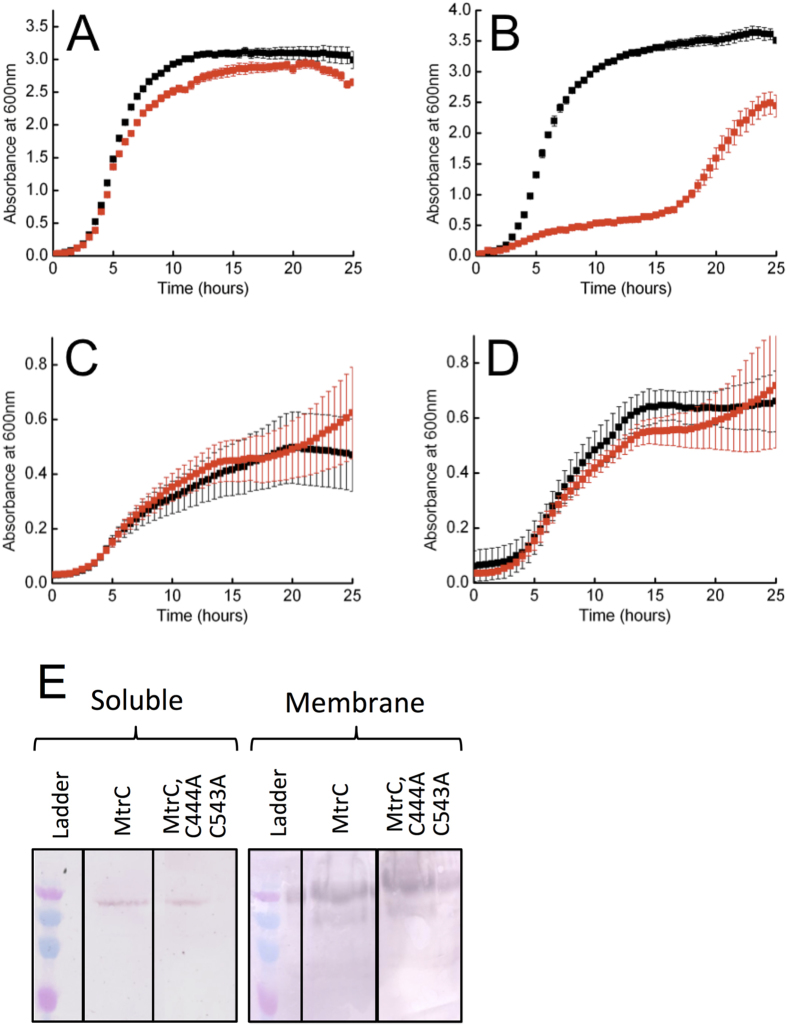
Growth rates and MtrC expression by *Shewanella oneidensis* LS661 complemented with plasmid pLS172 or pLS172-C444A,C453A (**A**) *S. oneidensis* LS661 transformed with pLS172 and grown aerobically in the presence of 1 μM FMN and either 0 mM arabinose (black squares) or 10 mM arabinose (red squares). (**B**) *S. oneidensis* LS661 transformed wih pLS172-C444A,C453A and grown aerobically in the presence of 1 μM FMN and either 0 mM arabinose (black squares) or 10 mM arabinose (red squares). (**C**) *S. oneidensis* LS661 transformed with pLS172 and grown anaerobically in the presence of 1 μM FMN and either 0 mM Arabinose (black squares) or 10 mM arabinose (red squares). (**D**) *S. oneidensis* LS661 transformed with pLS172-C444A,C453A and grown anaerobically in the presence of 1 μM FMN and either 0 mM arabinose (black squares) or 10 mM arabinose (red squares). The error bars show the standard deviation for 3 repeats of each experiment. (**E**) Western blot analyses of soluble and membrane solubilized fractions of *Shewanella oneidensis* LS661 expressing recombinant MtrC or MtrC_C444A,C453A_. Cells were induced with arabinose and separated into soluble and membrane fractions. Membrane fractions were subsequently solubilized using Triton X100. The presence of MtrC was detected using antibodies specific to the MtrC sequence position 399-410 and visualised using an alkaline phosphatase conjugated secondary antibody.

The aerobic growth of *S. oneidensis* LS661 pLS172 was the same in the presence and absence of the pLS172 inducer arabinose (Fig. 2A). In contrast, the aerobic growth profiles of *S. oneidensis* LS661 pLS172-C444A, C453A showed that, in the presence of arabinose, there was a significant lag phase of about 20 hours (Fig. 2B). The same *S. oneidensis* constructs were grown under anaerobic conditions using sodium fumarate as an electron acceptor (Fig. 2C,D). The anaerobic growth profiles of the two *S. oneidensis* constructs were similar in the presence and absence of arabinose, suggesting the extended lag phase observed under aerobic conditions was due to the loss of the CX_8_C motif from the membrane bound MtrC in the presence of oxygen. In order to determine the levels of expression for each MtrC isoform, the two different strains were grown anaerobically overnight using fumarate as a terminal electron acceptor and induced with 10 mM arabinose. After lysis, a Western blot using antibodies specific to MtrC revealed that both MtrC and MtrC-C444A, C453A were expressed in *S. oneidensis* LS661. The separated cell components show the major proportion of the MtrC was associated with the membrane fractions (Fig. 2E).

### **T**he MtrC CX_8_C motif forms a redox active disulfide that regulates flavin binding

**Figure 3:**
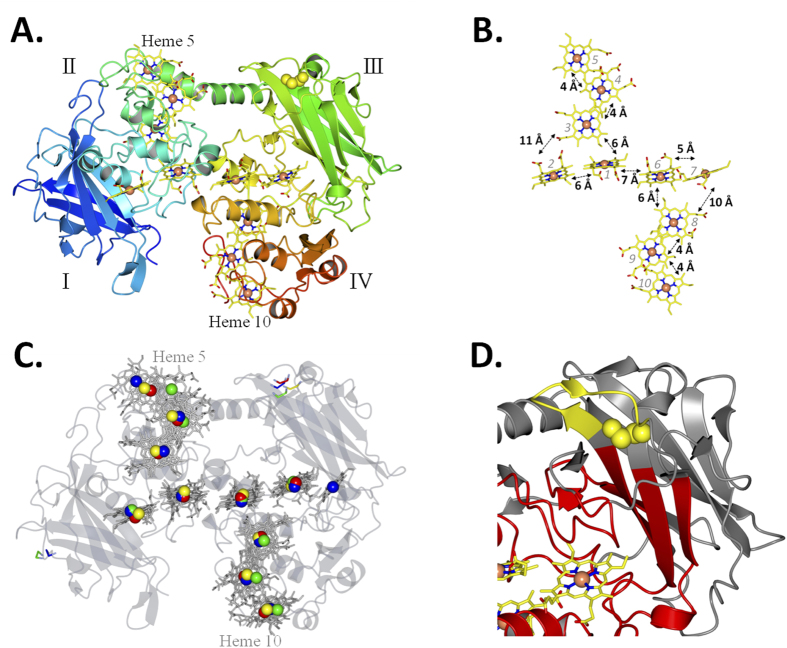
Crystal structure of MtrC at 1.8 Å resolution (PDB ID 4LM8). (**A**) Cartoon representation of MtrC. The four domains indicated by roman numerals. The polypeptide chain is shown in cartoon representation and coloured from blue (N-terminus) to red (C-terminus). The iron atoms of the hemes are represented as orange spheres and the porphyrin rings of the hemes are shown as yellow sticks. The cysteines of the disulfide bond are represented as yellow spheres. (**B**) Heme packing and putative electron transfer distances between porphyrin rings (**C**) Alignment of iron atoms from MtrC (yellow) over the iron atoms of MtrF (red), OmcA (green) and UndA (blue). The transparent structure of MtrC is shown in grey as (**D**) Domain III of MtrC showing the position of the disulfide (yellow spheres). Residues within 16 Å of heme 7 are shown in red.

To confirm the conformation of the CX_8_C motif we solved the x-ray crystal structure of *S. oneidensis* MR-1 MtrC (Fig. 3A, Table 1). MtrC has substantial structural homology to MtrF, UndA and OmcA with two multi-heme domains flanked by two β-barrel domains and the same staggered deca-heme cross present in all structures. The structure reveals that, with approximate dimensions of 90 Å × 60 Å × 40 Å, MtrC is too large to fit into a 28-strand MtrB barrel and so would likely be mostly exposed on the surface of the cell. This is in agreement with previous experiments showing MtrC could be completely digested from whole cells using proteinase K^11^. The ten hemes of MtrC are arranged in a ‘staggered-cross’ configuration that is a shared feature of the group of OMMC proteins (Fig. 3B). Alignment of the MtrC heme iron atoms with the iron atoms of UndA, MtrF and OmcA reveals that the heme arrangement of MtrC has a closer homology to MtrF with an r.m.s.d of 1.7 Å, than to UndA or OmcA with an r.m.s.d. of 2.1 Å (Fig. 3C). The most significant differences between the heme arrangement of all four structures are the extra heme 7 in UndA and the position of heme 5. The position of this heme is conserved between MtrC and MtrF, but is displaced in both UndA and OmcA. Both MtrC and MtrF are proposed to form tight porin-cytochrome MtrCAB or MtrFED complexes, while UndA and OmcA form more transient complexes. This could imply that the position of heme 5 is conserved in MtrC and MtrF as a result of complex formation, but has diverged in OmcA and UndA as formation of a tight complex is no longer required.

The conserved cysteines 444 and 453 of the CX_8_C motif form a single disulfide within domain III of MtrC. This disulfide is formed between a loop of 8 amino acids that contains a solvent exposed phenylalanine and valine (Fig. 3D). The N-terminal of the disulfide is positioned at the end of a long β-strand, while the C-terminal leads to a second loop of approximately 15 amino acids before forming a β-strand of the barrel. The majority of the side chains inside both domain I and III β-barrels are hydrophobic, and the surface of domain I contains mainly hydrophilic residues. However, in domain III there is a hydrophobic cleft next to heme 7 that contains three phenylalanines, which in the UndA is occupied by an extra heme.

**Figure 4:**
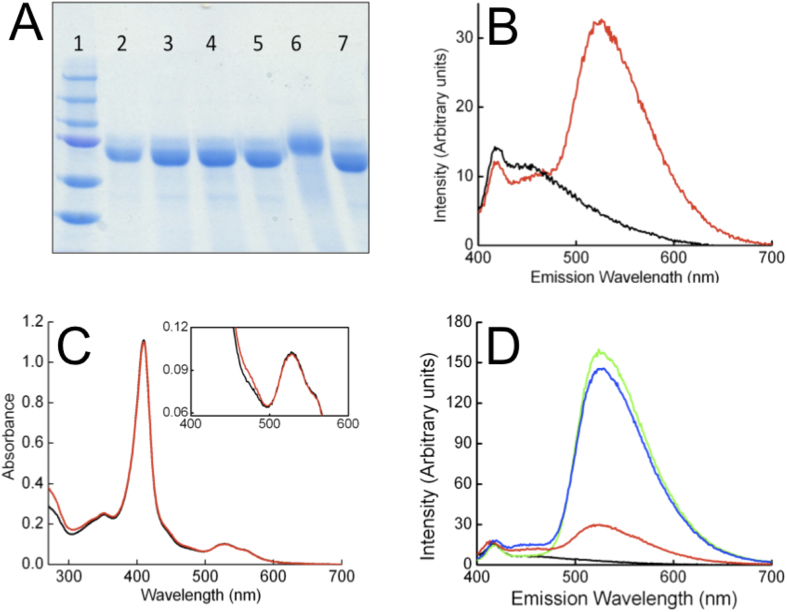
Formation of an MtrC-FMN complex in the presence of glutathione. (**A**) Coomassie stained SDS polyacrylamide gel of MtrC after incubation with glutathione and AMS. *(1)* Protein Markers of 250, 150,100, 75, 50 and 37 kDa molecular weight; *(2*, *7)* MtrC as isolated. *(3)* MtrC and 1 mM glutathione. *(4)* MtrC and 1 mM dithothreitol. *(5)* MtrC and 2 mM AMS. *(6)* MtrC, 1 mM glutathione and 2 mM AMS. (**B**) Anaerobic fluorescence spectrum of MtrC isolated from a solution containing 1 μM MtrC and 10 μM FMN in the presence (red line) and absence (black line) of 1 mM glutathione. (**C**) Absorbance spectra of MtrC isolated from a solution containing FMN and glutathione (red line) overlaid on a spectrum of oxidised MtrC (black line). *Inset:* The absorbance difference at 470 nm was use to measure a stoichiometry of 0.93 ± 0.05 FMN molecules per MtrC over three experiments. (**D**) Fluorescence spectra of anaerobic 0.4 μM MtrC (Black line); anaerobic 0.4 μM MtrC and 0.2 μM FMN (green line); 0.4 μM MtrC, 0.2 μM FMN, 1 mM glutathione before (red line) and after exposure to air (blue line).

Glutathione was used to reduce the single disulfide in MtrC, and the thiol-reactive reagent AMS (4-acetamido-4'-maleimidylstilbene-2,2'-disulfonic acid) was used to monitor the redox state of the MtrC cysteines as described previously^24^. In the absence of glutathione, isolated MtrC incubated with AMS migrated the same distance on SDS-polyacrylamide gels as MtrC alone. However, migration of MtrC was retarded after incubation with both glutathione and AMS, consistent with the ~500 Da AMS probe covalently attaching to the two thiol groups of the reduced cysteines (Fig. 4A). The UV-visible spectral properties of micromolar solutions of MtrC were not altered by incubation with 1 mM reduced glutathione under anaerobic conditions, demonstrating that incubation of MtrC with glutathione could reduce the redox-active MtrC disulfide but did not alter the redox state of the hemes.

MtrC was incubated with excess FMN in the absence and presence of 1 mM reduced glutathione under anaerobic conditions, and then passed through an anaerobic PD10 column. Examination of the eluted samples showed that the fluorescence spectrum (λ_ex_ 365 nm) of the glutathione incubated sample showed a peak at 525 nm that was absent from the sample without glutathione (Fig. 4B). The spectral feature at 525 nm arises from FMN and demonstrates that FMN associated specifically with MtrC in the presence of glutathione. The electronic absorbance spectrum of the MtrC-FMN complex showed that the hemes of MtrC remained oxidized after incubation with FMN and glutathione (Fig. 4C). Comparison of the spectrum of oxidized MtrC with that of the isolated MtrC-FMN complex revealed features that could be attributed to FMN (Fig. 4C inset). Using an ε_410_ of 1,260 mM^−1^ cm^−1^ for MtrC and an ε_470_ of 12.5 mM^−1^ cm^−1^ for FMN a stoichiometry of 0.93 ± 0.05 FMN molecules per MtrC molecule was determined, indicating that MtrC binds a single molecule of FMN. Similar experiments using riboflavin instead of FMN showed that MtrC was also capable of tightly binding a single molecule of riboflavin, and that riboflavin binding was dependent on the reduction of MtrC by glutathione (Supplemental figure 1A,B).

Monitoring fluorescence changes on anaerobic incubation of MtrC with FMN and glutathione, there was a 5-fold loss of fluorescence intensity due to quenching of the signal originating from the FMN isoalloxazine ring (Fig. 4D). This quenching, which did not occur when FMN alone was incubated with glutathione, was reversible, as exposure to air caused over 90% of the original fluorescence intensity to return, demonstrating that the interaction between MtrC and FMN was reversible on exposure to air.

### Thiol/disulfide regulated flavin binding was also observed for OmcA, MtrF and UndA

**Figure 5:**
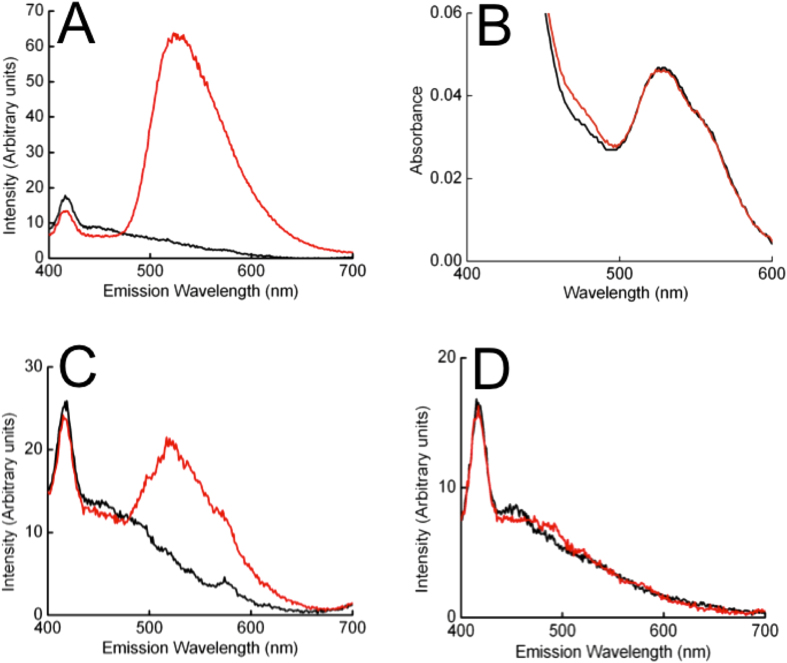
Fluorescence and UV-visible absorbance spectra of outer membrane multiheme cytochromes incubated with FMN and glutathione. (**A**) Anaerobic fluorescence spectra of OmcA isolated from a solution containing 10 μM FMN in the presence (red line) and absence (black line) of 1 mM glutathione. (**B**) Absorbance spectra of 1 μM OmcA isolated from a solution containing FMN and glutathione (red line) overlaid on a spectrum of oxidised OmcA (black line) (**C**) Anaerobic fluorescence spectrum of MtrF isolated from a solution containing 10 μM FMN in the presence (red line) and absence (black line) of 1 mM glutathione. (**D**) Anaerobic fluorescence spectrum of UndA isolated from a solution containing 10 μM FMN in the presence (red line) and absence (black line) of 1 mM glutathione.

Co-elution of FMN and OmcA was observed in the presence, but not the absence, of glutathione (Fig. 5A). It was evident from the absorbance spectrum of the isolated, anaerobic OmcA-FMN complex that both protein and FMN were present in their oxidised forms (Fig. 5B). Comparison of absorbances at 470 and 410 nm showed that the ratio of flavin to protein was approximately 0.95 FMN: 1 OmcA, as observed for MtrC. Similar results were also observed for OmcA with riboflavin (Supplemental figure 1C). As observed for MtrC-FMN, the fluorescence of the OmcA-FMN complex increased greatly on exposure to air, demonstrating the release of FMN upon thiol oxidation. OMMC-FMN interactions were also observed after incubation of MtrF or UndA with FMN and reduced glutathione (Fig. 5C,D) , although the fluorescence emission intensity at 525 nm was lower than that recorded for MtrC-FMN or OmcA-FMN, and the signal to noise ratio was higher due to background scattering effects.

## Discussion

While it has been clearly demonstrated that MtrCAB is capable of supporting electron transfer through direct contact, the role of flavins in facilitating extracellular electron transfer between cell and mineral surface is still poorly understood. It is clear that flavins play an important role in the reduction of Fe(III)oxide under anaerobic conditions in culture as the presence of flavin in the media, either secreted or artificially added, is required for optimal reduction^21^. After postulating that the redox state of MtrC may have an effect on the affinity of MtrC for FMN, we were able to isolate MtrC-flavin complexes after reduction of the MtrC disulfide. This MtrC flavocytochrome rapidly loses the flavin cofactor upon exposure to oxygen, presumably by causing the disulfide bond to reform. The closest heme group to the MtrC disulfide is heme 7, suggesting that FMN would associate somewhere on domain III, in close proximity to heme 7. This region on domain III of the OMMC’s has been previously suggested as a flavin-binding site^18^,^19^. In the fumarate reductase of *S. oneidensis* MR-1 the edge-to-edge distance between the flavin cofactor and the nearest heme group is 7.5 Å, and this distance would place the MtrC flavin binding site approximately halfway between heme 7 and the disulfide bond^25^. Interestingly, under aerobic conditions, *Paquete et al.* showed transient interactions with FMN and riboflavin for both MtrC and OmcA but no interaction was seen with MtrF or UndA^22^. In MtrC and OmcA there is a hydrophobic cleft next to heme 7 that is not observed in MtrF and in UndA is occupied by an extra heme. If the hydrophobic cleft next to heme 7 is important for flavin binding this might explain why under anaerobic, reducing conditions, we see stable flavin association with MtrC and OmcA and weaker association with MtrF and UndA.

These data provide a new role for *S. oneidensis* flavin secretion, in providing flavin for cofactor insertion into MtrC on the cell surface. The oxidising environment of the periplasm would prevent flavin insertion before transport through the outer membrane, while in the cytoplasm MtrC is expressed in an unfolded state before transport through the *sec* pathway. In order to overcome this *S. oneidensis* has generated a flavin export system that will allow flavin to be separately transported to the surface of the cell, where, under reducing conditions, it may bind to MtrC or OmcA. This implies that redox-controlled FMN binding to MtrC has an important function during *S. oneidensis* respiration. One possibility is that the bound FMN is involved in the two-electron reduction of a second, diffusible, FMN molecule. Several proteins involved in respiration and photosynthesis use a tightly bound organic cofactor to mediate two electron transfer to a loosely bound organic shuttle, including photosystem II and the cytochrome bc_1_ complex, and this may be another mechanism that allows coupled electron transfer to a flavin shuttle.

Previous work using anaerobic cultures showed a role for flavins in electron transfer to both iron oxides and electrodes. Under these anaerobic conditions it is likely that both MtrC and OmcA of *Shewanella oneidensis* are present as flavocytochromes and the increased iron reduction observed is caused in part by the presence of flavocytochromes on the surface of the protein. In contrast, aerobic growth of *S. oneidensis* would cause the exposed disulfides to reform and any bound flavin would be released into the medium. *Shewanella* are typically found in aquatic sediments at the oxic/anoxic interface, and this mechanism would allow them to instantly respond to any changes in the oxygen level of the sediment. The absence of a disulfide in MtrC expressed by *S. oneidensis* L661 pLS172-C444A, C453A means this modified MtrC would remain in the flavocytochrome form. These cells were unable to grow under aerobic conditions, suggesting catalytic reduction of di-oxygen by the MtrC flavocytochrome, forming reactive oxygen species such as superoxide and peroxide. The reduction of oxygen by flavoproteins is a well-studied phenomenon, and *S. oneidensis* has been shown to be susceptible to exposure to ionising radiation, at least partly due to the formation of reactive oxygen species^26^,^27^,^28^.

Taken together, these data lead to the hypothesis that, under anaerobic or sub-oxic conditions, *S. oneidensis* expresses OMMC flavocytochromes on the cell surface to facilitate electron transfer to extracellular acceptors such as soluble flavins or metal oxides. However, in the presence of oxygen, the OMMC flavocytochromes of *S. oneidensis* rapidly dissociate to prevent non-specific reduction that could ultimately result in cellular oxidative damage and compromise the viability of the cell.

### Experimental procedures

#### Expression and Purification of Outer Membrane Multiheme Cytochromes

Soluble OmcA, UndA and the detergent solublised form of MtrF were all isolated as described previously^17^,^18^,^19^. To obtain soluble MtrC the *mtrC* encoding gene was amplified from *S. oneidensis MR-1* and cloned into a pBAD 202 (*Invitrogen)* plasmid. MtrC was solubilized by replacing the 25 amino acids comprising the N-terminal signal peptide and acetylation site and replacing with the N-terminal signal peptide of MtrB of *S. oneidensis* MR-1 (MKFKLNLITLALLANTGLAVAADG). A V5-epitope/6xhis tag was added to the C-terminus (KGELKLEGKPIPNPLLGLDSTRTGHHHHHH). The *S. oneidensis* MR-1 strains LS329 containing pLS146 was grown aerobically at 30 °C in Terrific Broth (TB) media containing 30 μg mL^−1^ kanamycin. Expression of MtrC was induced by addition of 1 mM arabinose at the mid-log phase of growth. Cells were grown overnight and removed from the media by centrifugation. The clarified media was concentrated to ~400 mL using either a stirred Amicon pressure cell or Vivascience vivaflow 200 ultrafiltration cassette with 30,000 Dalton molecular weight cutoffs. The concentrated media was dialysed overnight using dialysis tubing with a 8,000 kDa molecular weight cut off against 5 litres of buffer containing 20 mM HEPES pH 7.8 in order to remove media components. Following the overnight dialysis the dialysis buffer was replaced with another 5 litres of 20 mM HEPES pH 7.8 and the protein was dialysed for a further 24 hours. The dialysed media/protein was centrifuged at 15,000 x g for 15 minutes to remove any precipitate before loading onto a 200 ml DEAE column pre-equilibrated with 20 mM HEPES pH 7.8. The column was washed with 20 mM HEPES pH 7.8 until a stable UV baseline was observed. The protein was eluted with a gradient of 0–500 mM NaCl over 850 mL and 10 mL fractions were collected. Fractions were analysed by SDS-PAGE staining with Coomassie. Fractions containing MtrC were pooled and concentrated to 10 mg mL^−1^ and buffer-exchanged into 20 mM HEPES pH 7.8 using a centrifugal concentrator with a 30,000 dalton molecular weight cutoff.

#### Phylogenetic analysis of MtrC cytochromes

Amino acid sequences of homologues of *Shewanella oneidensis* MR-1 MtrC were retrieved by BLAST search (blast.ncbi.nlm.nih.gov)^29^. Sequences were aligned utilizing the Clustal Omega server^30^. An average distance phylogenetic tree based upon percentage identity was calculated using the Jalview sequence analysis software^31^. Calculated trees were formatted and a figure prepared utilizing the interactive tree of life tool (itol.embl.de)^32^.

#### Cloning and expression of MtrC C444A,C453A

Plasmid pLS172 containing the *mtrC* gene was transformed into the *S. oneidensis* LS661 Δ*mtrC* strain as described previously^33^,^34^. pLS172 is a pBAD202/D-TOPO® plasmid vector containing the *mtrC* gene, an arabinose inducible promoter and a kanamycin resistance gene for selection. To mutate the *mtrC* codons responsible for Cys_444_ and Cys_453_ two rounds of site directed mutagenesis were performed.

Initially primer 5'-GTAGGTTGGTCAATGGCTTCTAGCGAAGGTAAG-3' and its complement primer were used as templates during PCR to mutate pLS172 into pLS172-C444A. This plasmid was then used in a second PCR round with primer 5'-TAAGTTTGTAGACGCTCAGACCCCTGCA-3' and complement primer to give pLS172-C444A,C453A. This was transformed into *S. oneidensis* LS661 to give *S. oneidensis* LS661 pLS172-C44A,453 A containing a plasmid based copy of *mtrC* with codons for both Cys_444_ and Cys_453_ mutated to alanines. The sequences of both plasmids were verified through nucleotide sequencing (Eurofins) after each PCR round.

#### Growth Studies of Shewanella oneidensis

Both *S. oneidensis* strains were grown aerobically overnight in 10 ml Luria broth (LB) containing 30 μg/mL kanamycin. These were used to provide 100 μl inoculum for 10 ml LB media stocks containing 30 μg/ml kanamycin, 1 μM FMN, 1 μM riboflavin and 10 mM arabinose. Samples were taken and used to fill wells in a 48 well transparent plate. The plate was incubated at 30 °C in a FLUOstar Optima microplate reader (BMG Labtech) with aerobic conditions achieved by shaking at 400 rpm. Repeat experiments were carried out under anaerobic conditions. 10 mL LB media stocks containing 30 μg/ml kanamycin, 1 μM FMN, 1 μM riboflavin and 50 mM sodium fumarate were inoculated with 100 μl inoculum from overnight cultures as before. Samples of each culture were aliquoted into a 48 well PCR plate before being transferred into a glove box with O_2_ maintained below 2 ppm and leaving for 30 min for O_2_ to dissipate out of the wells and culture solution. A transparent cover was then laid over the wells before the plate lid was glued into place using an airtight adhesive. The plate was then removed from the glove box and incubated in the plate reader under conditions stated above but without agitation before each read to help maintain anaerobic conditions. A control of inoculated LB lacking sodium fumarate was included on the plate to confirm anaerobic conditions.

#### MtrC crystallisation and data collection

Crystals of MtrC were obtained from a sitting-drop vapour diffusion setup with 0.2 M sodium acetate pH 5.0, 0.1 M CaCl_2_ and 21% PEG 6,000 as the reservoir solution. Crystals formed in both 1:1 and 2:1 (reservoir:protein) drops with a total drop volume of 0.6 μl. Crystals were cryo-protected by transferring to a solution of 0.2 M sodium acetate pH 5.0, 0.1 M CaCl_2_, 21% PEG 6,000 and 20% ethylene glycol before being vitrified by plunging into liquid nitrogen. Data were collected on MtrC crystals in a gaseous stream of nitrogen at 100 K on beamlines I02 and I04-1 at the Diamond Light Source (UK). MtrC crystals were of space group P2_1_2_1_2_1_ with typical cell dimensions of a = 52.91 b = 89.77 c = 153.90 Å. A SAD dataset was collected at a wavelength of 1.72 Å to a final resolution of 3.2 Å. Further datasets from single crystals were collected using an x-ray wavelength of 0.97 Å.

#### MtrC structure determination and refinement

MtrC datasets were processed using XIA2, or MOSFLM and SCALA as part of the CCP4 package^35^,^36^. The SAD dataset of MtrC was analysed using the autosol pipeline within the PHENIX software suite^37^. The program HySS located 10 heavy atom sites and the electron density maps calculated with PHASER/RESOLVE were sufficiently interpretable to manually place ten hemes corresponding to a single MtrC molecule in the asymmetric unit.

The autobuild program ARPWARP^38^ was used to build residues 45-670 followed by alternating rounds of manual building and refinement using PHENIX^37^ or REFMAC^39^. The final model was refined to an Rcryst (Rfree) value of 16.6 (20.3) %. This model has no residues in the disallowed region of the Ramachadran plot. Coordinates have been deposited in the RCSB Protein Data bank under accession code 4LM8.

#### Spectroscopic investigations of flavin binding to OMMC

Stock solutions of OMMC and either FMN or riboflavin were purged with nitrogen gas and taken into an anaerobic glove box (Belle Technology). Solid reduced glutathione was taken into an anaerobic glove box and dissolved in oxygen free 20 mM HEPES pH 7.6 buffer just before use. Under anaerobic conditions, in a foil wrapped container, the OMMC solution was diluted to 2.5 ml with 20 mM HEPES pH 7.6; flavin and glutathione added and the mixture incubated at room temperature for 30 min. The mixture was then applied to a foil-covered, anaerobic PD10 column. On elution with 20 mM HEPES pH 7.6, the first 1 ml of the protein fraction was transferred to a cuvette, anaerobically sealed and retained for spectroscopic analysis.

For fluorescence quenching experiments, OMMC, flavin and glutathione mixtures were prepared anaerobically in a fluorescence cuvette that was anaerobically sealed and incubated for 10 min before spectroscopic analysis. After 5 min, for a given set of conditions, repeated scans were performed to check the fluorescence intensity did not change.

#### SDS polyacrylamide gel electrophoresis of MtrC after incubation with glutathione and AMS

In an anaerobic glove box, different combinations of the reagents reduced-glutathione and 4-acetamido-4'-maleimidylstilbene-2,2'-disulfonic acid (AMS) were added to aliquots from a 50 μM stock of MtrC. These were incubated anaerobically for 30 minutes then 10μl applied to a 10% SDS PAGE gel. The gel was run at 30 mA for 2 hours then stained with Coomassie based staining solution.

## Additional Information

**How to cite this article**: Edwards, M. J. *et al.* Redox Linked Flavin Sites in Extracellular Decaheme Proteins Involved in Microbe-Mineral Electron Transfer. *Sci. Rep.* **5**, 11677; doi: 10.1038/srep11677 (2015).

## Supplementary Material

Supplementary Information

## Figures and Tables

**Table 1:** Data collection and refinement statistics for MtrC.

	**MtrC-SAD**	**MtrC-Native**
**Data collection**		
Space group	P 2_1_ 2_1_ 2_1_	P 2_1_ 2_1_ 2_1_
Cell dimensions		
*a*, *b*, *c* (Å)	52.91, 89.77, 153.90	53.12, 90.44, 154.34
α, β, γ(°)	90.00, 90.00, 90.00	90.00, 90.00, 90.00
Resolution (Å)	89.8-3.2(3.3-3.2)	58.7-1.8 (1.9-1.8)
*R*_sym_ or *R*_merge_	17.6% (38.3%)	8.7% (38.8%)
*I* / σ*I*	27.5 (15.3)	12.0 (3.4)
Completeness (%)	100 (100)	97.6 (97.6)
Multiplicity	25.6(25.4)	4.8 (4.5)
**Refinement**		
Resolution (Å)		1.80
No. reflections		67724
*R*_work_ / *R*_free_		0.17/0.20
No. atoms		
Protein		4719
Ligand/ion		435
Water		936
*B*-factors		
Protein		17.7
Ligand/ion		12.7
Water		30.2
R.m.s. deviations		
Bond lengths (Å)		0.016
Bond angles (°)		1.2

^*^Values in parentheses are for highest-resolution shell.
