# Supplementary material for: Redox Linked Flavin Sites in Extracellular Decaheme Proteins Involved in Microbe-Mineral Electron Transfer
Source: Sci Rep. 2015 Jul 1;5:11677. doi: 10.1038/srep11677 (PMC4486940; doi:10.1038/srep11677)
Supplement: Supplementary Information [file srep11677-s1.doc]

**Supporting Information: Redox Linked Flavin Sites in Extracellular Decaheme Proteins Involved in Microbe-Mineral Electron Transfer.**

Marcus J Edwards^1§^, Gaye White^1§^, Michael Norman^1^, Alice Tome-Fernandez, Emma Ainsworth^1^ Liang Shi^2^, Jim K Fredrickson^2^, John M Zachara^2^, Julea Butt^1^, David J Richardson^1*^, Thomas A Clarke^1*^

^1^Centre for Molecular and Structural Biochemistry, School of Biological Sciences and School of Chemistry, University of East Anglia, Norwich NR4 7TJ, United Kingdom

^2^Pacific Northwest National Laboratory, Richland, WA 99352, USA

^§^These two authors contributed equally to this work.

*For correspondence e-mail tom.clarke@uea.ac.uk and d.richardson@uea.ac.uk


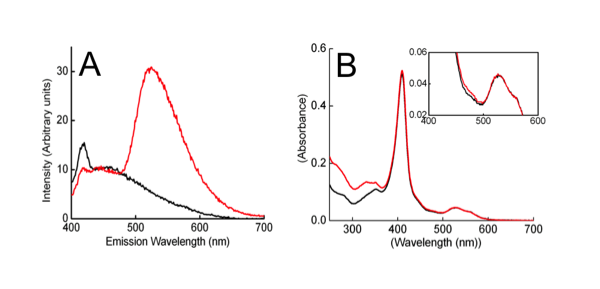
Supplemental figure S1. **(A**) Anaerobic fluorescence spectra of MtrC isolated from a solution containing 1 µM MtrC and 10 µM riboflavin in the presence (red line) and absence (black line) of 1 mM glutathione. **(B)** Absorbance spectra of 1 µM MtrC isolated from a solution containing 10 µM riboflavin and 1 mM glutathione (red line) overlaid on a spectrum of oxidised MtrC (black line) **(C)** Anaerobic fluorescence spectrum of OmcA isolated from a solution containing 1 µM OmcA and 10 µM riboflavin in the presence (red line) and absence (black line) of 1 mM glutathione.

**
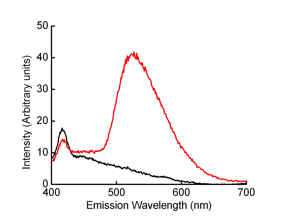
**

C
